# Genetic Diversity and Population Structures in Chinese Miniature Pigs Revealed by SINE Retrotransposon Insertion Polymorphisms, a New Type of Genetic Markers

**DOI:** 10.3390/ani11041136

**Published:** 2021-04-15

**Authors:** Cai Chen, Xiaoyan Wang, Wencheng Zong, Enrico D’Alessandro, Domenico Giosa, Yafen Guo, Jiude Mao, Chengyi Song

**Affiliations:** 1 College of Animal Science & Technology, Yangzhou University, Yangzhou 225009, China; chencai9596@hotmail.com (C.C.); wxyan@yzu.edu.cn (X.W.); zongzone@outlook.com (W.Z.); 2 Department of Veterinary Science, University of Messina, 98168 Messina, Italy; edalessandro@unime.it; 3 Department of Clinical and Experimental Medicine, University Hospital of Messina, 98125 Messina, Italy; dgiosa@unime.it; 4 College of Animal Science and Technology, Guangxi University, Nanning 530005, China; guoyafen@163.com; 5 Bond Life Science Center, University of Missouri, Columbia, MO 65211, USA; maoj@missouri.edu

**Keywords:** retrotransposon insertion polymorphism, genetic markers, SINE, miniature pig, Chinese Bama miniature pig

## Abstract

**Simple Summary:**

Our previous studies suggested that the short interspersed nuclear element (SINE) retrotransposon insertion polymorphisms (RIPs), as a new type of molecular marker developed very recently, are ideal molecular markers and have the potential to be used for population genetic analysis and molecular breeding in pigs and possibly it can be extended to other livestock animals as well. However, no report is available for the application of SINE RIPs in population genetic analysis in livestock, including pigs. Here, we evaluated 30 SINE RIPs in several indigenous Chinese miniature pig breeds, including three subpopulations of Bama pigs (BM-cov, BM-clo, and BM-inb). BM-cov is a subpopulation conserved in the national conservation farm, and BM-clo is a closed population maintained over 30 years with only 2 boars and 14 sows imported from its original area, while BM-inb herd is an 18 generation continuous inbreeding line based on the BM-clo population. To our knowledge, it is the first time to report the genetic diversity, breed differentiation, and population structures for these populations by using SINE RIPs, and which suggests the feasibility of SINE RIPs in pig genetic analysis.

**Abstract:**

RIPs have been developed as effective genetic markers and popularly applied for genetic analysis in plants, but few reports are available for domestic animals. Here, we established 30 new molecular markers based on the SINE RIPs, and applied them for population genetic analysis in seven Chinese miniature pigs. The data revealed that the closed herd (BM-clo), inbreeding herd (BM-inb) of Bama miniature pigs were distinctly different from the BM-cov herds in the conservation farm, and other miniature pigs (Wuzhishan, Congjiang Xiang, Tibetan, and Mingguang small ear). These later five miniature pig breeds can further be classified into two clades based on a phylogenetic tree: one included BM-cov and Wuzhishan, the other included Congjiang Xiang, Tibetan, and Mingguang small ear, which was well-supported by structure analysis. The polymorphic information contents estimated by using SINE RIPs are lower than the predictions based on microsatellites. Overall, the genetic distances and breed-relationships between these populations revealed by 30 SINE RIPs generally agree with their evolutions and geographic distributions. We demonstrated the potential of SINE RIPs as new genetic markers for genetic monitoring and population structure analysis in pigs, which can even be extended to other livestock animals.

## 1. Introduction

Miniature pigs, due to their physiological, anatomical, and genetic similarities to human beings and the relative easy handling, are regarded as a key animal model in biomedical studies [1,2]. There are several indigenous miniature pig breeds in China, such as Xiang, Wuzhishan, Bama, Mingguang small-ear, and Tibetan. All of them originated in the mountainous areas of the south or south-west, far away from the mainland of China [3,4,5]. The Congjiang Xiang pig, a subpopulation of Xiang pig breed, originated in Guizhou province, while the Wuzhishan pigs originated from the Wuzhishan Mountains in Hainan Island [3], and Mingguang small-ear pigs in Tengchong, Yunan Province. Tibetan pigs, which originated on the Tibetan Plateau, were adapted to a high-altitude and a low-temperature environment, distributed across Sichuan, Gansu, Yunnan, and Tibet of China. The Bama miniature pig breed formed in an isolated Bama Yao Autonomous County of Guangxi Zhuang Autonomous Region, south-west of China [4]. Currently, there are three subpopulations kept in two conservation farms seated in Guangxi province. The national conservation farm located at Bama County of Guangxi Zhuang Autonomous Region has conserved one population, which was named as BM-cov, while one closed herd (BM-clo) and one highly inbred (BM-inb) line are kept at Guangxi University [4]. All these breeds are characterized by early sexual maturity, good disease resistance, and strong adaptability in local environments [4,5,6]. In addition, because of isolation from the outside and the long time of natural and artificial selections, inbreeding has continuously increased in these populations, and the genetic diversities are expected to decrease significantly compared with the other Chinese local pig populations [4,7]. However, the genetic diversity, breed differentiation, and population structures in these populations still remain largely unknown. 

Retrotransposons, as major genomic parasites of mammals, occupying for 30–45% of the genomic sequences in mammals [8,9,10,11,12,13], which can be classified into three major groups: long interspersed nuclear elements (LINEs), short interspersed nuclear elements (SINEs), and endogenous retroviruses (ERVs) [14]. It has been found that some retrotransposons are active, and they can be mobile in the host genome and generate insertion polymorphisms within a specific population [15,16,17]. Due to their ubiquitous distribution and high copy number in the genome, it is believed that RIPs are suitable for genetic marker development for potential use in population genetic analysis [18]. This is particularly true for SINEs, which are the second most abundant retrotransposons in the genomes of most mammals but represent the most extensive distribution in genomes due to their small size [11,15,19] Furthermore, SINE RIPs have been suggested as “nearly ideal” genetic markers [20]. The primate SINE (Alu) insertion polymorphisms, as genetic markers, have been extensively applied for population genetic analysis in human beings [21]. Our recent studies revealed that SINEs account for 11.05% of the pig genome [22], which are evenly distributed in chromosomes, and large-scale SINE RIPs (over 10,000) have been identified in the dog genome [19,23], thus, a similar prediction could be expected for the pig genomes. The objectives of the present study were to assess the SINE RIPs as a new type of molecular markers in terms of polymorphic information content and heterozygosity in pigs that have evolved recently. Moreover, the genetic diversity, differentiation, and population-relationship among the BM-inb, BM-clo, and BM-cov was studied by applying RIPs and comparing with the other miniature pigs (Xiang, Wuzhishan, Mingguang small-ear, Tibetan), an Italian native pig breed and Landrace, which is used as an outgroup control because of their distant origin from China and no potential intercross with Chinese miniature pigs. Our data provided an important validation of the SINE RIPs in the genetic monitoring and population structure analysis, suggesting their application potential in genetic analysis and molecular breeding in pigs, and even livestock, since most livestock share a similar mobilome landscape.

## 2. Materials and Methods

### 2.1. Animals and DNA Isolation

**Figure 1:**
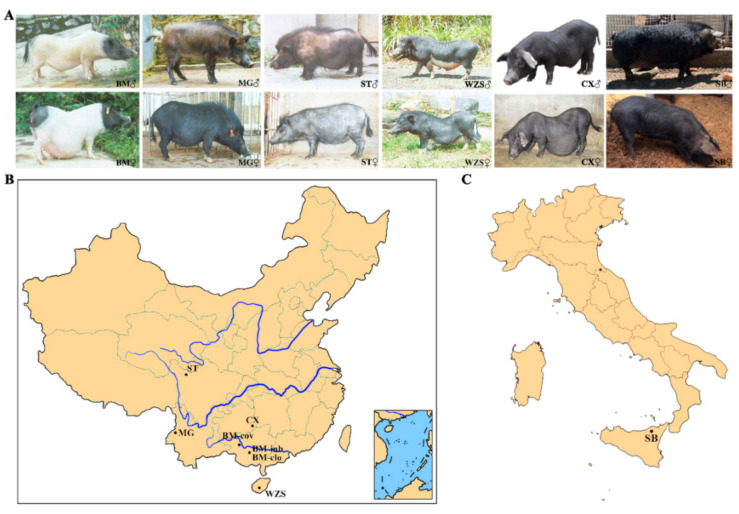
The photos and geographical distribution of miniature pigs and Sicilian black pig. (**A**) The photos of miniature pigs and Sicilian black pig. (**B**) Geographical distributions of the seven miniature pig populations in China. (**C**) Geographical distributions of Sicilian black pigs in Italy. BM: Bama miniature pig, MG: Mingguang small ear pig, ST: Tibetan pig in Sichuan province, WZS: Wuzhishan pig, CX: Congjiang Xiang pig, SB: Sicilian black pig. BM-clo, BM-inb, and BM-cov are three subpopulations of Bama pigs, kept in the national conservation farm (BM-cov), a closed herd (BM-clo), and a highly inbred line (BM-inb) in Guangxi University. The abbreviation for pig applies to all figures and tables.

Ear or blood samples were collected from seven miniature pig populations of BM-cov, BM-clo, BM-inb, Congjiang Xiang, Wuzhishan, Tibetan, Mingguang small ear, and one Italian pig breed (Sicilian black pig/Nero Siciliano pig) and one commercial breed (Landrace pig), with a sample size of 24, 29, 22, 28, 24, 28, 20, 32, and 32, respectively. The TIANamp Genomic DNA Kit (TIANGEN Biotech Co. Ltd., Beijing, China) was used for DNA isolation from the samples of each animal using the TIANamp Genomic DNA Kit (TIANGEN Biotech Co. Ltd., Beijing, China). The quality of DNA was verified by using NanoPhotometer (Implen, Munich, Germany) and electrophoresis. BM-cov samples were taken from the national Bama conservation farm in Bama autonomous county, Guangxi Zhuang autonomous region. BM-clo and BM-inb samples were collected from the farm in Guangxi University pig farms in Nanning, Guangxi province. Congjiang Xiang samples were provided by Guizhou University pig farm in Guiyang, Guizhou province. Wuzhishan samples were from the national Wuzhishan conservation farm in Haikou, Hainan province. Tibetan samples were from the pig farm in the Animal Husbandry Research Institute of Ganzi Tibetan Autonomous Prefecture, Sichuan province. Mingguang small ear pig samples were taken from the national Mingguang small ear pig conservation farm in Tengchong, Yunnan Province. Sicilian black pig is an autochthonous genetic type that lives in the woods of the Nebrodi and Madonie mountains on the northern coast of the Mediterranean island of Sicily (Italy) [24]. The Landrace pig samples, used as a positive outbreed control, were taken from the breeding farm in Xuzhou China. The photos of six local breeds were shown in Figure 1A. The geographical distribution of seven miniature pig populations was shown in Figure 1B, and the Sicilian black pigs were shown in Figure 1C.

### 2.2. Development of RIP Makers

SINE RIPs were identified based on our recently established protocol (unpublished data). Briefly, the main process was divided into four main steps. (1) Screening SINE insertions in the genomes with a custom library which was built in advance [22] by using RepeatMasker. (2) The flanking sequences of these SINE insertions in the nonreference genomes were mapped to the reference genome using Blat [25], thereby, each insertion’s information corresponding to the reference genome was obtained from each nonreference genome. (3) The differential insertions, designated as putative SINE insertion polymorphisms were obtained using a bedtools window. (4) The putative SINE RIPs were manually verified by local BLAST [26] and PCR amplification. In the current study, a total of 36 SINE RIPs in each chromosome were randomly selected for PCR evaluation. Out of 36 total SINE RIPs, 30 SINE RIPs showed clear polymorphic bands across the seven miniature pig populations by PCR analysis. PCR primers were designed according to the 5′ and 3′ flanking sequences of SINE insertion sites and synthesized by TSINGKE Biological Technology co., Ltd. (TSINGKE, Nanjing, China). All primer sequences and information are listed in Table S1.

PCR reactions were carried out in a total volume of 20 μL, composed of 1 μL 50 ng/μL genomic DNA, 10 μL 2× Taq Master Mix buffer (Vazyme, Nanjing, China), 1 μL of 10 μM primer F, 1 μL of 10 μM primer R, and 7 μL water. The PCR reaction conditions were set as following: 94 °C for 5 min for an initial denaturation, followed by 30 cycles (94 °C for 30 s, 58 °C for 30 s, 72 °C for 1 min) and a final extension of 10 min at 72 °C. The PCR products were detected by electrophoresis in a 1.5% agarose gel with 1× TAE buffer using a constant voltage of 130 V for 30 min. Gels were stained by ethidium bromide and visualized with UV fluorescence.

### 2.3. Statistics and Population Genetic Analyses

Allele frequencies, number of effective alleles per locus (Ne), observed heterozygosity (Ho), expected heterozygosity (He), fixation index (F, including F_IS_, F_ST_, F_IT_), and the Hardy–Weinberg equilibrium test was determined using Popgene [27] (version 1.32). The polymorphic information content (PIC) was calculated according to the formula: $PIC=1-\sum_{i=1}^{n}p_{i}^{2}-\sum_{i=1}^{n-1}\sum_{j=i+1}^{n}2p_{i}^{2}p_{j}^{2}$, where n was the number of alleles, pi was the frequency of the insertion allele in the population, and pj was the frequency of the deletion allele in the population.

Cluster analysis based on Nei’s genetic distance [28] was carried, and a UPGMA tree was constructed by Mega7 [29]. Based on the results of SINE RIPs, we performed principal component analysis (PCA) using the R statistics package (v. 3.6.3). The population structure of the seven miniature pig groups were established using the Bayesian clustering method in STRUCTURE [30] (version 2.3.4). Further, Delta K values were calculated and the appropriate K value was estimated by implementing the method of Evanno et al. (2012) using the STRUCTURE Harvester [31] program (http://taylor0.biology.ucla.edu/struct_harvest/, 22 January 2021). CLUMPP [32] (version 1.1.2) and Distruct [33] (version 1.1) were used to repeat sampling, analyzing, and drawing.

## 3. Results

### 3.1. Evaluation of SINE RIPs in Chinese Miniature Pig Populations

**Figure 2:**
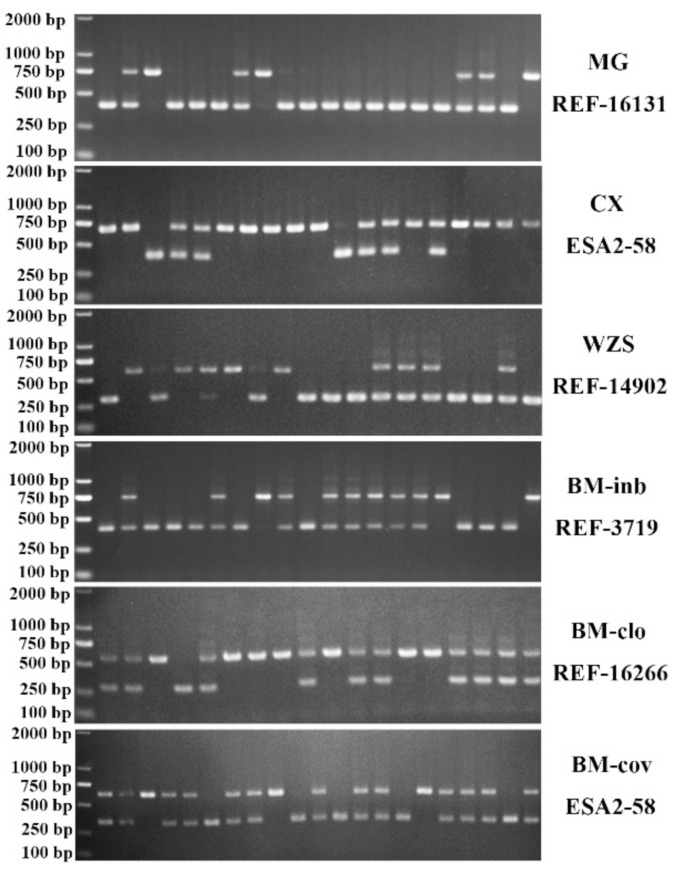
A representative electropherogram of SINE RIPs used for detection in the pig populations. MG: Mingguang small ear pig, WZS: Wuzhishan pig, CX: Congjiang Xiang pig, BM-clo, BM-inb, and BM-cov are three subpopulations of Bama pigs, kept in the national conservation farm (BM-cov), a closed herd (BM-clo) and a highly inbred line (BM-inb) in Guangxi University. REF-16131, ESA2-58, REF-14902, REF-3719, REF-16266 were SINE RIP name.

Thirty-six SINE RIP genetic markers (two SINE RIPs in each chromosome), which were predicted according to the protocol described in methods, were selected to evaluate their polymorphisms in 243 animals of seven Chinese miniature pig breeds, one commercial breed, and one Italian native breed (Sicilian black pig) which was selected as an outbreed control. The genomic coordinates of these markers, their PCR primers, and the predicted PCR product sizes were listed in Table S1. Thirty RIPs displayed polymorphism in these miniature pig populations and were used for further population genetic analysis. Six RIP markers were monomorphism in these miniature pigs and not used for the present study (Table S1). The representative PCR detection results of these RIPs are shown in Figure 2. Their PCR detection results of the final thirty RIPs are summarized in Figure S1. These RIPs were biallelic with clear and stable amplified bands. Based on these data, three genotypes were identified: the first with a single small band of homozygous type absent SINE insertion defined as SINE^−/−^, with band size ranging from 273 to 450 bp in length; the second, a single large band of homozygous type with SINE insertion named as SINE^+/^^+^, with PCR product sizes ranging from 415 to 739 bp in length, and the third heterozygote type named SINE^+/−^ with both small and large bands. For thirty RIP markers, the inbreeding pig population of BM-inb displayed very low inbreed diversity and only three RIP markers were polymorphic. Low inbreed diversity was also observed for Landrace and the closed herd of Bama miniature pigs, where 15 and 17 RIP markers were polymorphic, respectively; while Bama miniature pigs at the conservation farm displayed similar inbreed diversity to other miniature pig breeds and the Italian pig breed. In total, 20, 30, 26, 23, 30, and 25 polymorphic RIPs were detected in BM-cov, Congjiang Xiang, Wuzhishan, Sichun Tibetan, Mingguang small ear, and Sicilian black pigs, respectively (Table 1). The genotype and allele frequencies of these RIPs and the Hardy–Weinberg equilibrium test for each RIP in each breed were summarized in Table 1 and Table S2. Significant variations of SINE insertion/deletion allele frequencies across these breeds were observed. However, most RIP insertion/deletion alleles in both BM-clo and BM-inb tend to be fixed (13 RIPs) or predominant (13 RIPs, >0.80 or <0.20). In addition, Sicilian black pigs displayed different insertion/deletion allele frequency distributions for the majority of RIPs compared to the Chinese miniature pig breeds (Table 1). The Hardy–Weinberg equilibrium analysis revealed that BM-inb, Tibetan, and Landrace showed a genetic equilibrium at all polymorphic loci. One RIP (REF-8430) in BM-clo pigs, six RIPs (REF-14427, REF-3719, REF-9435, ESA1-98, REF11172, and REF3992) in Congjiang Xiang, three RIPs (REF-3719, REF-4531, and REF-10096) in BM-cov, four RIPs (REF-14427, REF-21609, REF-3719, and REF16266) in Wuzhishan pigs, two RIPs (REF-16131 and REF17668) in Mingguang small ear pigs, three RIPs (REF-13104, ESA1-25, and REF-9432) in Sicilian black pigs were in genetic disequilibrium (Table 1 and Table S2).

### 3.2. Genetic Diversity of China Miniature Pig Populations Revealed by SINE RIPs

The genetic parameters, including Ne, He, Ho, PIC, and Fis for each population, are presented in Table 2. The average Ne among the nine populations was 1.3481, ranging from 1.0542 to 1.5813. The average PIC among nine populations was 0.1736, ranging from 0.0263 to 0.2708, of which the Mingguang small ear population was the highest, while BM-inb was the lowest. The average He among nine populations was 0.2139, ranging from 0.0333 (BM-inb) to 0.3477 (Mingguang small ear). A similar variance pattern for Ho was observed in these breeds. BM-inb had the lowest genetic diversity represented by lowest Ne, He, Ho, and PIC values. BM-clo had the second-lowest diversity. These results further confirmed that inbreeding reduces genetic diversity, and the BM-inb pigs had been inbred for many years, and most loci were homozygous. The genetic diversity of the BM-clo also decreased significantly due to limited bloodlines for mating within the subpopulation. While the genetic diversity of BM-cov was similar to the other Chinese miniature pig breeds, the estimates of Ho and He were relatively higher in Chinese Congjiang Xiang, Wuzhishan, Tibetan, Mingguang small ear, BM-cov than those of the Italian breed of Sicilian black, and Landrace. Mingguang small ear population displayed the highest genetic diversity among the eight investigated populations.

**Figure 3:**
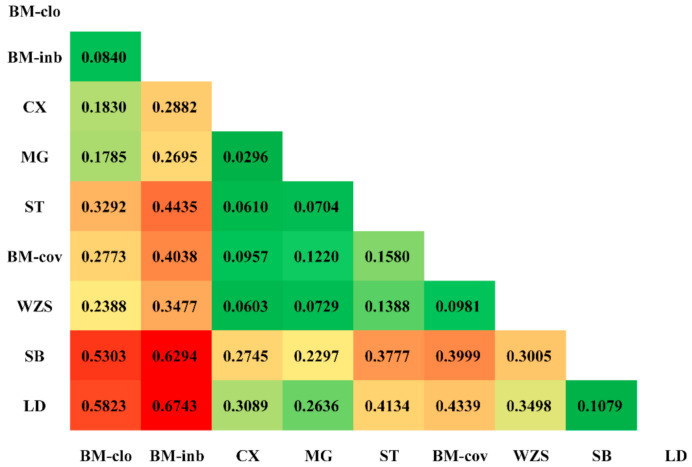
Heatmap of the fixation indices (F_ST_) between miniature pigs. The higher F_ST_ estimated is in red, the lower F_ST_ estimated is in green.

For genetic differences among populations, the F_ST_ value was 0.3780 when all loci were considered, indicating that approximately 37.80% of the total genetic variation between breeds, while the remaining 62.20% were attributed to differences among individuals within a breed (Table 1). For each individual locus, this value ranged from 0.0273 (REF-5597) to 0.8800 (REF-13182). The heatmap of the pairwise Fst values among these populations is shown in Figure 3. Overall, the BM-clo and BM-inb populations showed a relatively higher degree of distance from the BM-cov compared to Congjiang Xiang, Mingguang small ear, Wuzhishan, Tibetan, and BM-cov. Estimates of the BM-clo and BM-inb against the BM-cov were 0.2773 and 0.4038, respectively, while the average differentiations among other miniature pigs (Congjiang Xiang, Mingguang small ear, Wuzhishan, Tibetan, and BM-cov) was 0.0907 ± 0.0376. Both BM-clo and BM-inb showed a relatively high genetic difference against other miniature pig populations as well as the Italian breed and Landrace. The genetic difference between BM-inb and Landrace was highest (Fst = 0.6743) and followed by the BM-inb and Sicilian black pair comparison (Fst = 0.6294).

The inbreeding coefficient, as evaluated by the F_IS_ parameter, averaged −0.0241 for all loci, and ranged from −0.3508 (REF-17668) to 0.2612 (REF-13182) (Table 1). A very high inbreeding coefficient (Fis >0.1) was found in Wuzhishan (0.0397) and BM-cov (0.0299) pigs (Table 2). However, as expected, the BM-inb, BM-clo, and Landrace had low Fis scores with 27, 13, and 14 SINE RIPs being homozygous in these two populations.

### 3.3. Genetic Distances between Chinese Miniature Pig Populations Based on SINE RIPs

The pairwise Nei’s distances between populations are shown in Table 3. The genetic distance among the seven miniature pig populations was relatively low (≤0.27), ranging from 0.01 to 0.27 by RIPs score, while the Sicilian black and Landrace, which were included as an outbreed heterotic group for the genetic distance computations, showed large distances from all miniature pig populations, indicating a great difference of these breeds from the Chinese miniature pigs. The smallest genetic distance (0.01) obtained was between the BM-Clo and the BM-inb, indicating a very low divergence of these varieties. However, unexpected large genetic distances (0.13) were also obtained between the subpopulations of BM breed (BM-cov, BM-inb, and BM-clo). BM-cov, as a subpopulation of Bama miniature pigs, has relatively high genetic distances from the BM-clo (0.16) and BM-inb (0.21), but relatively small genetic distances from the other miniature pig breeds, ranging from 0.07 when compared with Congjiang Xiang and Wuzhishan to 0.11 with Mingguang small ear pigs and Tibetan pig. On the other hand, both BM-clo and BM-inb have relatively small genetic distances from the Congjiang Xiang and Mingguang small ear breeds, but large genetic distances from BM-co, whereas, the average genetic distance between pairs was 0.04 among Congjiang Xiang, Tibetan, and Mingguang small ear pigs. The Congjiang Xiang had the lowest distances from the rest of the Chinese miniature pigs, ranging from 0.03 when compared with Mingguang small ear pigs to 0.07 with BM-cov, except BM-inb (0.17), and BM-clo (0.12).

### 3.4. Population Structure of Chinese Miniature Pigs Revealed by SINE RIPs

**Figure 4:**
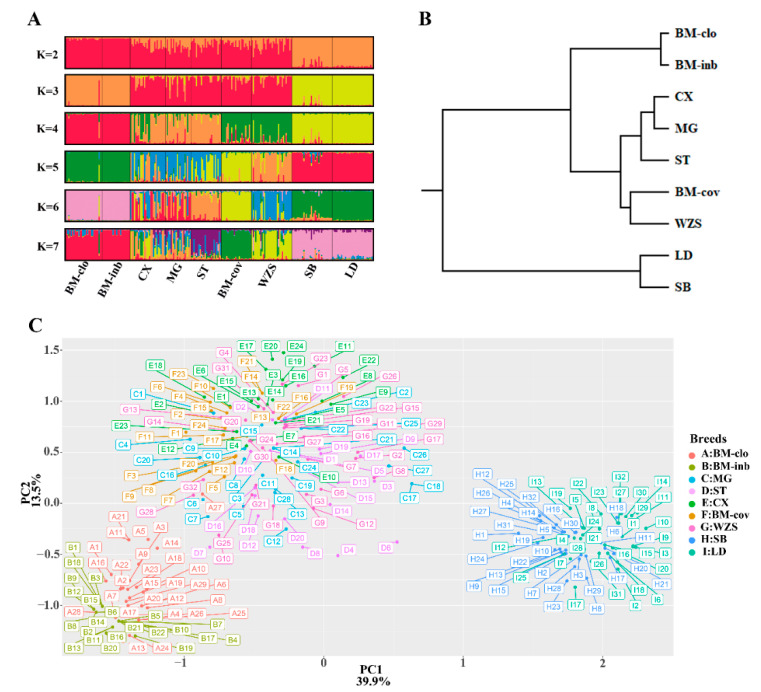
Population structure of seven Chinese miniature pigs and Sicilian black pigs. (**A**) Graphical representation of the results generated by Structure software with K 2–7. (**B**) UPGMA tree on Nei’s genetic distances. (**C**) PCA plot for eight pig populations; distribution along the first two eigenvectors.

To measure the population structure and degree of admixture, we applied the STRUCTURE algorithm and principal component analysis (PCA), and the UPGMA tree was generated based on the Nei’s genetic distance. We analyzed the grouping situation when K ranged from 2 to 7, meaning that we presupposed that all individuals originated from K ancestors or breeds. The cluster results based on STRUCTURE are shown in Figure 4A. Interestingly, BM-clo and BM-inb were separated from the other breeds when K = 2, and they lacked any affinity with Chinese miniature pigs, even BM-cov. When K = 3, the European pigs (Sicilian black) and Landrace were separated from Chinese miniature pigs and formed three distinct ancestries; Congjiang Xiang, Mingguang small ear, Tibetan, and BM-cov had large proportions of common ancestry. This agrees with the results of the PCA and the UPGMA tree analyses, which placed the BM-inb and BM-clo as a distinct cluster from the other miniature pigs (Congjiang Xiang, Mingguang small ear, Tibetan, BM-cov, and Wuzhishan), and the outbreed Sicilian black pigs and Landrace pigs (Figure 4B,C). A particular feature at K = 4 is that Congjiang Xiang, Mingguang small ear, Tibetan were separated completely from BM-cov and Wuzhishan. BM-cov breed clearly shares a common ancestry with the Wuzhishan breed, which also agrees with the UPGMA tree analysis. BM-cov and Wuzhishan had a tendency to group in a new subclade, while Congjiang Xiang, Mingguang small ear, and Tibetan also tend to cluster in the same subclade (Figure 4C). BM-cov separated from Wuzhishan when K ≥ 5. Progressively, as K increased, the contributions of the assumed populations resulted in the complete separation of the seven breeds.

## 4. Discussion

Active retrotransposons move randomly in the genome, resulting in different types of structural variations, such as insertion, deletion, reversion, and recombination [34], and may influence the nearby gene activities and result in the variations of phenotypes [35]. Thus, the genetic markers based on the RIPs are suggested as an important tool for studies of genetic diversity, and evolution, QTL mapping, and even for molecular breeding in plants [36,37,38,39]. RIPs have been developed and efficiently applied for genetic analysis in animals, such as ERV RIPs in sheep [40], deer [41], chicken [38], mice [42], and disease analysis in humans [43,44]. In pigs, the impact of retrotransposons on lncRNA and protein-coding genes have been systematically evaluated, and over 80% of genes contained retrotransposon insertions, and about half of protein-coding genes (44.30%) and one-fourth (24.13%) of lncRNA genes contained the youngest retrotransposon insertions [22], which are putative polymorphic insertions and may contribute to genetic and phenotypic variations across breeds. Two cases of phenotypic variations associated with L1 RIPs were reported in pigs previously [45,46], and a recent study identified eight L1 RIPs in pigs, and one of them was significantly associated with economic traits [47]. Three SINE RIPs were reported in the pig Vertnin gene, one SINE RIP was suggested as a putatively causative mutation of vertebral number variation [48,49]. One SINE insertion in the first intron of the PDIA4 gene was associated with the litter size of the pig [50]. These data suggested that genetic and phenotypic variations caused by RIPs seem common in pigs, and they may play roles in population differentiation and breed formation. In the present study, 36 SINE RIPs, which were predicted based on the recently developed protocol (unpublished data), were used to evaluate the genetic diversity and population structure among seven miniature pig populations; 83% of them (30/36) were confirmed to be polymorphic by PCR, indicating that the established SINE RIP screening protocol is highly reliable. Furthermore, high-quality bands were obtained when the PCR products were designed between 500–700 bp in sizes, and the validity of the markers of SINE RIPs was also well-supported by the genetic parameter estimates.

Genetic markers based on the microsatellites are widely used for the analysis of genetic diversity of Chinese miniature pigs, and the values of He and PIC are designated as important genetic parameters of genetic diversity [51]. Botstein [52] proposed that the loci with PIC >0.5 are highly informative based on the microsatellite makers, loci with a PIC value between 0.25 and 0.5 are moderately informative, while loci with PIC <0.25 are low informative value. Wang et al. used 32 microsatellite markers to analyze the genetic diversity of miniature pigs [53], and found the PIC values of Bama, Guizhou Xiang pig, and Tibetan pig were 0.5469, 0.7296, and 0.7663, respectively; Min et al. [54] and Yao et al. [55] used microsatellites to evaluate the genetic diversity of Wuzhishan pigs and found the means of PIC were 0.7069 and 0.84, respectively. In another report, the PICs of Tibetan, Xiang, Wuzhishan, and Diannan small-ear pigs were estimated as 0.696, 0.552, 0.653, and 0.585, respectively [56]. These data suggested that the genetic markers based on the microsatellites, in most miniature pig populations, were highly informative, and these breeds display high genetic diversity. However, the PIC values of seven miniature pig populations ranged from 0.0263 to 0.2708 estimated by using the SINE RIPs, which are substantially lower than the PICs estimated based on the microsatellite markers. This is because SINE RIP markers are biallelic, while microsatellite markers are multiple-allelic. Ho values in Bama (0.21), Wuzhishan (0.25), and Tibetan (0.24) estimated based on 1.4 million SNP chip [57] are generally similar to our estimations for Bama (0.2097 ± 0.1974), Wuzhishan (0.2698 ± 0.1999), and Tibetan (0.2431 ± 0.2021), which are listed in Table S3. These data indicate again that SINE RIPs are reliable and applicable in genetic analysis, with advantages of low costs, easy handling, and genotyping compared with SNP chip and microsatellite markers.

Based on the SINE RIPs, we also found that most investigated miniature pigs (Congjiang Xiang, Mingguang small ear, Wuzhishan, Tibetan, and BM-cov) displayed relatively high genetic diversity compared with Sicilian black and Landrace pigs according to the genetic parameters (He, Ho, PIC); while the BM-inb and BM-clo represented low genetic diversity, which generally agrees with the known genetic background and histories of these two populations. The BM-clo population, kept as a closed population in Guangxi University farm for over 30 years, was originally set up by importing 14 sows and 2 boars from the original place (Bama town) in 1987, while the BM-inb population, offspring of the 10th generation of BM-clo pigs, is a highly inbred line due to continuous inbreeding (>18 generations). However, the large genetic distances of BM-inb and BM-clo from the BM-cov pigs disagreed with their population relationships since both BM-inb and BM-clo populations were originated from the BM-cov. The exact reason is not apparent, but it may be because most detected loci (30 SINE RIPs) have been highly homogeneous in BM-inb and BM-clo pigs due to inbreeding, which resulted in an inaccurate estimation of genetic distances and population structures. The low Fis value estimation in Bov-inb may be due to the same reason. It was clear that BM-inb and BM-clo shared a large proportion of ancestry. But they did not show a close genetic relationship with the BM-cov breed. BM-cov breed was an admixture with Wuzhishan in the same clade, and Congjiang Xiang, Tibetan, and Mingguang small ear pigs formed a distinct clade, which is in good agreement with the phylogenetic analysis of 47 Chinese and European domestic breeds and wild boars based on 1.4 million SNP chip [57]. Bama and Wuzhishan display a very close phylogenetic relationship in the same branch, while Congjiang Xiang with other local pig breeds also cluster in the same clade but with a distinct phylogenetic position from Bama and Wuzhishan. In addition, low genetic distances between populations of Congjiang Xiang, Tibetan, and Mingguang small ear pigs indicated a small divergence of these breeds and that they may share the common ancestors. The inclusion of Sicilian black and Landrace pigs as outgroup in the PCA, UPGMA tree, and STRUCTURE analysis well-supported the genetic relationship among the seven Chinese miniature pig populations.

In summary, we identified 30 SINE RIP markers and applied them to determine the genetic diversity, differentiation, and population structure in seven Chinese miniature pig populations. Low genetic diversity, large genetic distance, and differentiation of BM-inb and BM-clo from the BM-cov and other miniature pig populations were observed. Our data revealed that the genetic distance, diversity, and breed-relationships between these populations generally agree with the evolutions and geographic distributions of these populations, and also basically agree with the population genetic analysis based on the SNP array, indicating that the SINE RIPs are reliable and applicable for population genetic analysis in pigs. In addition, our data also suggested that more SINE RIPs are required for population genetic analysis for high inbreeding populations. Overall, we demonstrated the potential of SINE RIPs in population genetic analysis, suggesting an alternative genetic marker that is simple, reliable, and high-quality. If RIP markers are analyzed in low numbers, it has the advantage of requiring no highly sophisticated instruments necessary to the capillary electrophoresis of labeled microsatellites or reading SNP chips. When a larger number of RIPs are analyzed, a labor-saving approach for genotyping is expected to be developed.

## Figures and Tables

**Table 1:** Polymorphism of insertion sites in seven Chinese pig populations.

SINE RIP	Insertion Frequency									No. of Populations Show Nonpolymorphic	No. of Populations Show Polymorphic/Not Comply with the Hardy–Weinberg Equilibrium	F_IS_	F_ST_
	MG	ST	WZS	CX	BM-cov	BM-clo	BM-inb	SB	LD				
REF-12270	0.25	0.19	0.25	0.43	0.15	0.00	0.00	0.52	1.00	3	6/0	−0.1407	0.4029
REF-13182	0.98	1.00	0.94	0.93	1.00	1.00	1.00	0.05	0.00	5	4/1	0.2612	0.8800
REF-14427	0.65	0.19	0.30	0.36	0.00	0.81	1.00	0.02	0.00	3	6/2	0.1356	0.5287
REF-16131	0.25	0.31	0.39	0.16	0.00	0.00	0.00	0.00	0.00	5	4/1	0.0255	0.2057
REF-21609	0.05	0.00	0.50	0.18	0.10	0.17	0.07	0.00	0.11	2	7/1	−0.2946	0.1812
REF-2929	0.33	0.23	0.28	0.04	0.48	0.02	0.00	0.25	0.33	1	8/0	−0.0630	0.1429
REF-3719	0.10	0.00	0.00	0.14	0.19	0.41	0.32	0.00	0.00	4	5/2	0.2028	0.1856
REF-4531	0.10	0.35	0.14	0.20	0.23	0.02	0.00	0.19	0.13	1	8/1	0.0242	0.0827
REF-5597	0.03	0.02	0.02	0.07	0.06	0.00	0.00	0.06	0.00	3	6/0	−0.0583	0.0273
REF-7445	0.10	0.58	0.00	0.04	0.00	0.00	0.00	0.06	0.00	5	4/0	−0.1497	0.4030
REF-8430	0.03	0.02	0.13	0.14	0.56	0.03	0.00	0.02	0.33	1	8/1	−0.1065	0.2665
REF-9435	0.35	0.13	0.11	0.39	0.02	1.00	1.00	0.03	0.00	3	6/1	−0.0393	0.6400
REF-10096	0.20	0.21	0.05	0.16	0.21	0.00	0.00	0.20	0.13	2	7/1	0.0502	0.0639
REF-11062	0.38	0.52	0.67	0.64	0.90	0.98	1.00	0.02	0.00	2	7/0	0.0181	0.5204
ESA1-98	0.18	0.00	0.16	0.13	0.00	0.00	0.00	0.88	1.00	5	4/1	−0.0300	0.7135
REF-11172	0.25	0.06	0.28	0.29	0.21	0.00	0.00	0.81	0.91	2	7/1	−0.1501	0.4540
REF-13104	0.73	1.00	0.66	0.96	1.00	0.98	1.00	0.03	0.42	3	6/1	−0.0407	0.5510
REF-14902	0.23	0.27	0.20	0.16	0.54	0.03	0.00	0.02	0.25	1	8/0	−0.0329	0.1643
REF-16266	0.58	1.00	0.70	0.89	1.00	0.66	0.70	0.14	0.00	3	6/1	−0.0179	0.4738
REF-17668	0.50	0.42	0.75	0.57	0.81	0.90	1.00	0.00	0.00	3	6/1	−0.3508	0.4746
ESA2-58	0.80	0.85	0.59	0.80	0.46	0.98	1.00	0.95	0.75	1	8/0	−0.0192	0.1813
DR-93949	0.33	0.15	0.08	0.25	1.00	0.00	0.00	0.88	0.92	3	6/0	−0.0583	0.5751
ESA1-16	0.38	0.10	0.92	0.46	0.81	1.00	1.00	0.94	1.00	3	6/0	−0.0558	0.5097
REF-3992	0.45	0.81	0.50	0.55	0.13	0.00	0.00	0.20	0.63	2	7/1	−0.0048	0.3262
ESA1-25	0.78	0.96	0.55	0.77	0.85	0.29	0.00	0.73	0.81	1	8/1	−0.0407	0.3652
ESA2-18	0.53	0.52	0.08	0.32	0.40	0.57	1.00	0.58	0.81	1	8/0	0.0011	0.2546
ESA1-33	0.28	0.00	0.00	0.14	0.00	0.12	0.00	0.53	1.00	5	4/0	−0.1187	0.5752
REF-9432	0.48	0.54	0.14	0.54	0.40	0.98	1.00	0.22	0.34	1	8/1	0.1906	0.3239
ESA1-42	0.65	0.75	0.70	0.79	0.52	0.19	0.00	1.00	1.00	3	6/0	0.1874	0.4350
ESA1-43	0.10	0.04	0.00	0.14	0.00	0.00	0.00	0.75	0.36	4	5/0	−0.0475	0.4312
No. of loci show nonpolymorphic	0	7	4	0	10	13	27	5	15	N	N	N	N

Note: MG: Mingguang small ear pig, ST: Tibetan pig in Sichuan province, WZS: Wuzhishan pig, CX: Congjiang Xiang pig, SB: Sicilian black pig. LD: Landrace pig, BM-clo, BM-inb, and BM-cov are three subpopulations of Bama pigs, kept in the national conservation farm (BM-cov), a closed herd (BM-clo), and a highly inbred line (BM-inb) in Guangxi University.

**Table 2:** Genetic parameters generated by the 30 SINE RIPs in all eight pig breeds.

Breed Name	Sample Size	He	Ho	Polymorphic Information Content (PIC)	Effective Number of Allele (Ne)	Fis
MG	20	0.3477 ± 0.1530	0.3550 ± 0.1997	0.2708 ± 0.1074	1.5813 ± 0.3184	−0.0520 ± 0.2803
ST	24	0.2378 ± 0.1926	0.2431 ± 0.2021	0.1885 ± 0.1445	1.3879 ± 0.3621	−0.0440 ± 0.1668
WZS	32	0.2814 ± 0.1783	0.2698 ± 0.1999	0.2238 ± 0.1319	1.4635 ± 0.3477	0.0397 ± 0.2956
CX	28	0.3181 ± 0.1409	0.3190 ± 0.1811	0.2543 ± 0.0975	1.5133 ± 0.3080	−0.0148 ± 0.2699
BM-cov	24	0.2252 ± 0.1998	0.2097 ± 0.1974	0.1777 ± 0.1503	1.3730 ± 0.3787	0.0299 ± 0.2626
BM-clo	29	0.1179 ± 0.1705	0.1253 ± 0.1841	0.1267 ± 0.2106	1.1858 ± 0.3025	−0.0174 ± 0.2803
BM-inb	22	0.0333 ± 0.1118	0.0333 ± 0.1126	0.0263 ± 0.0866	1.0542 ± 0.1884	−0.0374 ± 0.1841
SB	32	0.1923 ± 0.1718	0.1958 ± 0.1859	0.1575 ± 0.1313	1.2955 ± 0.3136	−0.0024 ± 0.2594
LD	32	0.1712 ± 0.1942	0.1813 ± 0.2057	0.1367 ± 0.1505	1.2780 ± 0.3427	−0.0845 ± 0.1309
Average	27	0.2139 ± 0.0988	0.2147 ± 0.0980	0.1736 ± 0.0744	1.3481 ± 0.1649	−0.0203 ± 0.0395

**Table 3:** Nei’s unbiased measures of genetic identity and genetic distance generated by the 30 SINE RIPs in eight pig populations.

Population Name	BM-clo	BM-inb	CX	MG	ST	BM-cov	WZS	SB	LD
BM-clo	-	0.99	0.89	0.88	0.80	0.85	0.85	0.59	0.53
BM-inb	0.01	-	0.84	0.85	0.76	0.81	0.81	0.55	0.50
CX	0.12	0.17	-	0.98	0.96	0.93	0.95	0.75	0.72
MG	0.13	0.16	0.03	-	0.95	0.90	0.93	0.79	0.75
ST	0.23	0.27	0.05	0.06	-	0.89	0.89	0.68	0.64
BM-cov	0.16	0.21	0.07	0.11	0.11	-	0.93	0.66	0.63
WZS	0.16	0.21	0.05	0.07	0.11	0.07	-	0.74	0.69
SB	0.52	0.59	0.29	0.24	0.38	0.42	0.30	-	0.95
LD	0.63	0.70	0.33	0.29	0.44	0.46	0.36	0.05	-

Nei’s genetic identity (above diagonal) and genetic distance (below diagonal).

## Data Availability

All data needed to evaluate the conclusions in this paper are present either in the main text or the supplementary materials.

## Supplementary Materials

The following are available online at https://www.mdpi.com/article/10.3390/ani11041136/s1, Figure S1 PCR detection results of all thirty RIPs, Table S1: Primers of the 36 SINE RIP markers, Table S2: The genotype frequency and Hardy–Weinberg equilibrium test for each RIP in nine populations. Table S3: Comparison of population genetic parameter results based on RIPs, microsatellites and SNPs.
