# Supplementary material for: Genetic Diversity and Population Structures in Chinese Miniature Pigs Revealed by SINE Retrotransposon Insertion Polymorphisms, a New Type of Genetic Markers
Source: Animals (Basel). 2021 Apr 15;11(4):1136. doi: 10.3390/ani11041136 (PMC8071531; doi:10.3390/ani11041136)

Congjiang Xiang pig

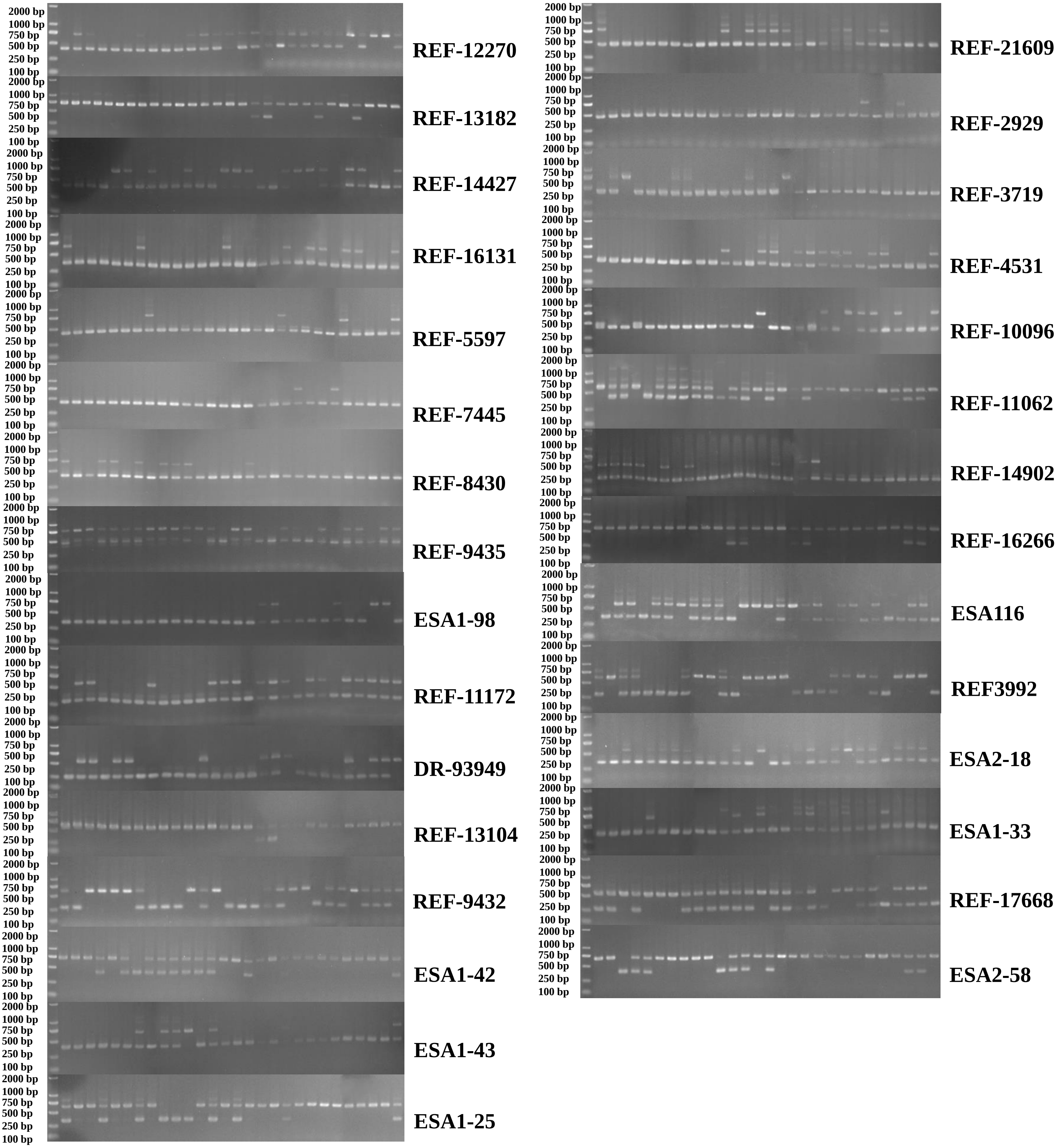

Mingguang Small ear pig

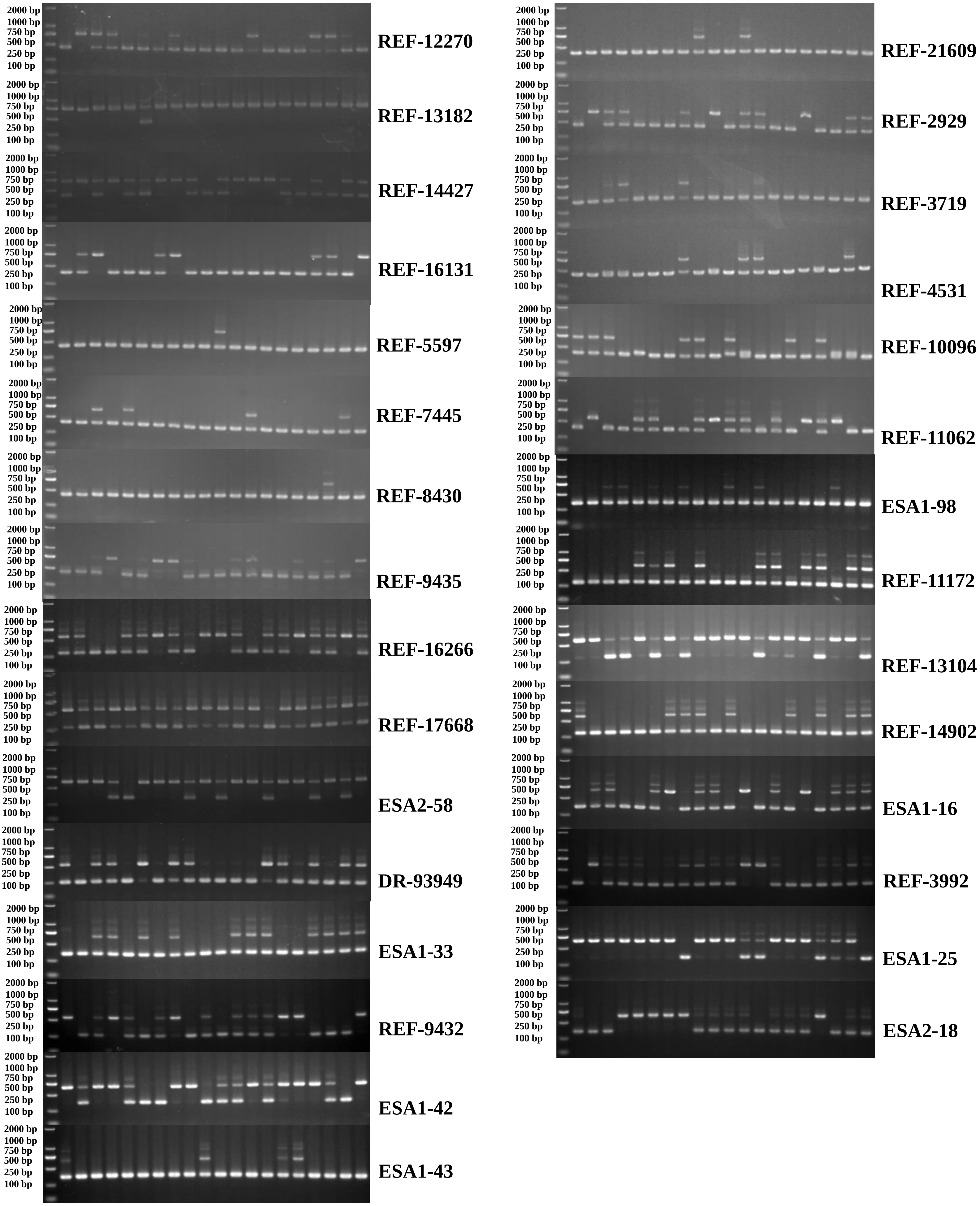

Bama pig inbred line

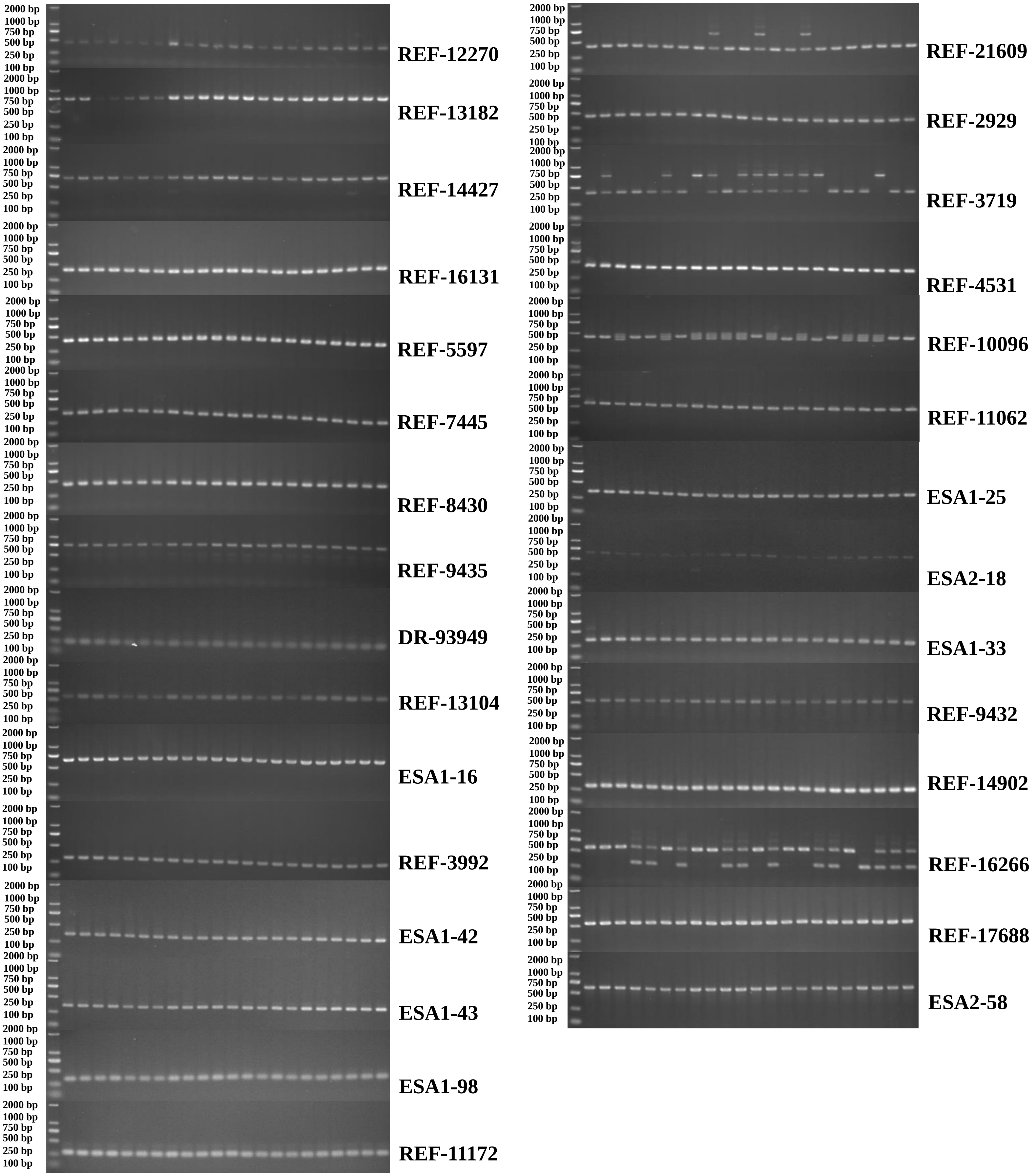

Bama pig closed herd

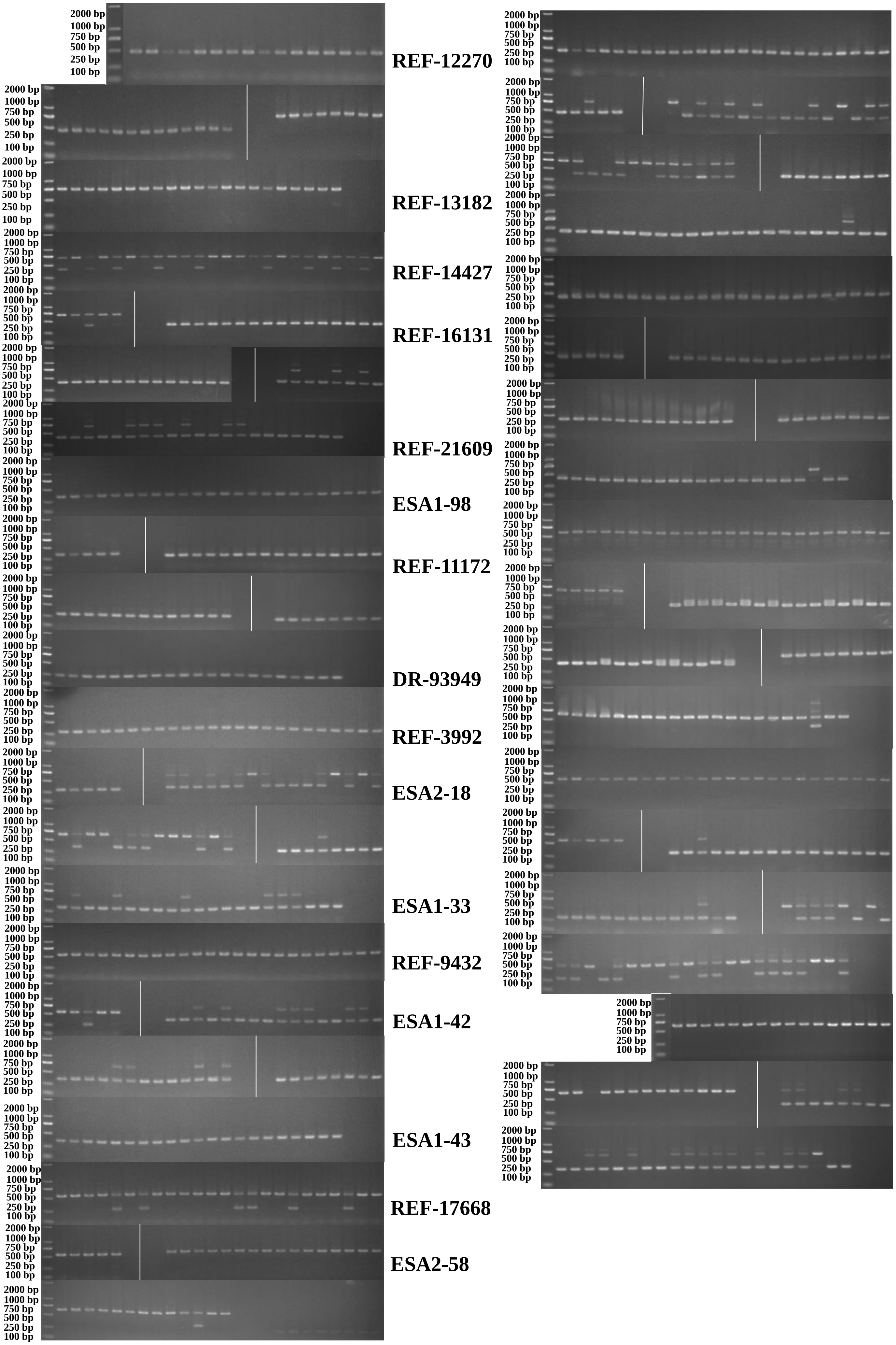

Sichuan Tibetan

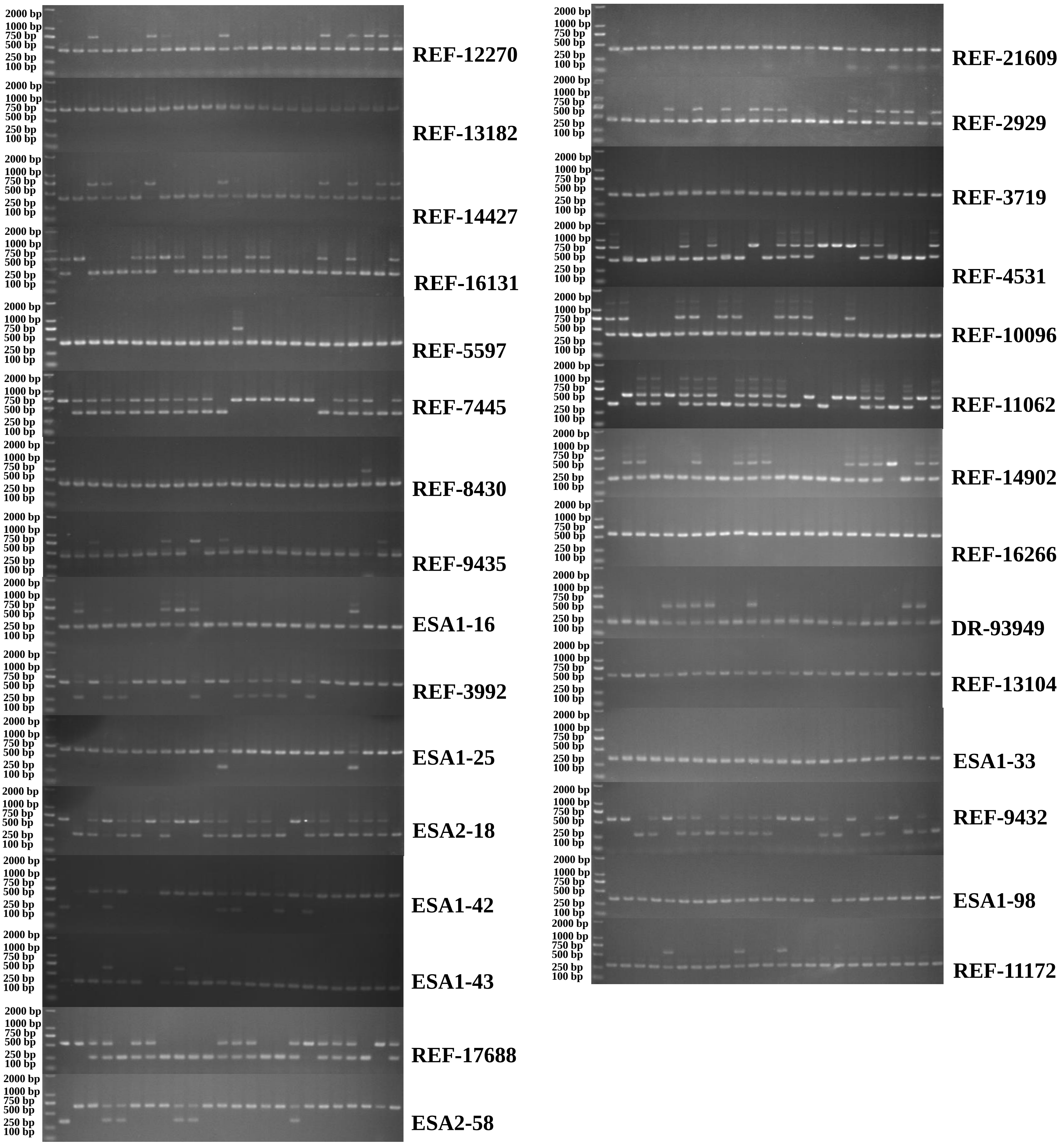

Bama located at conservation farm

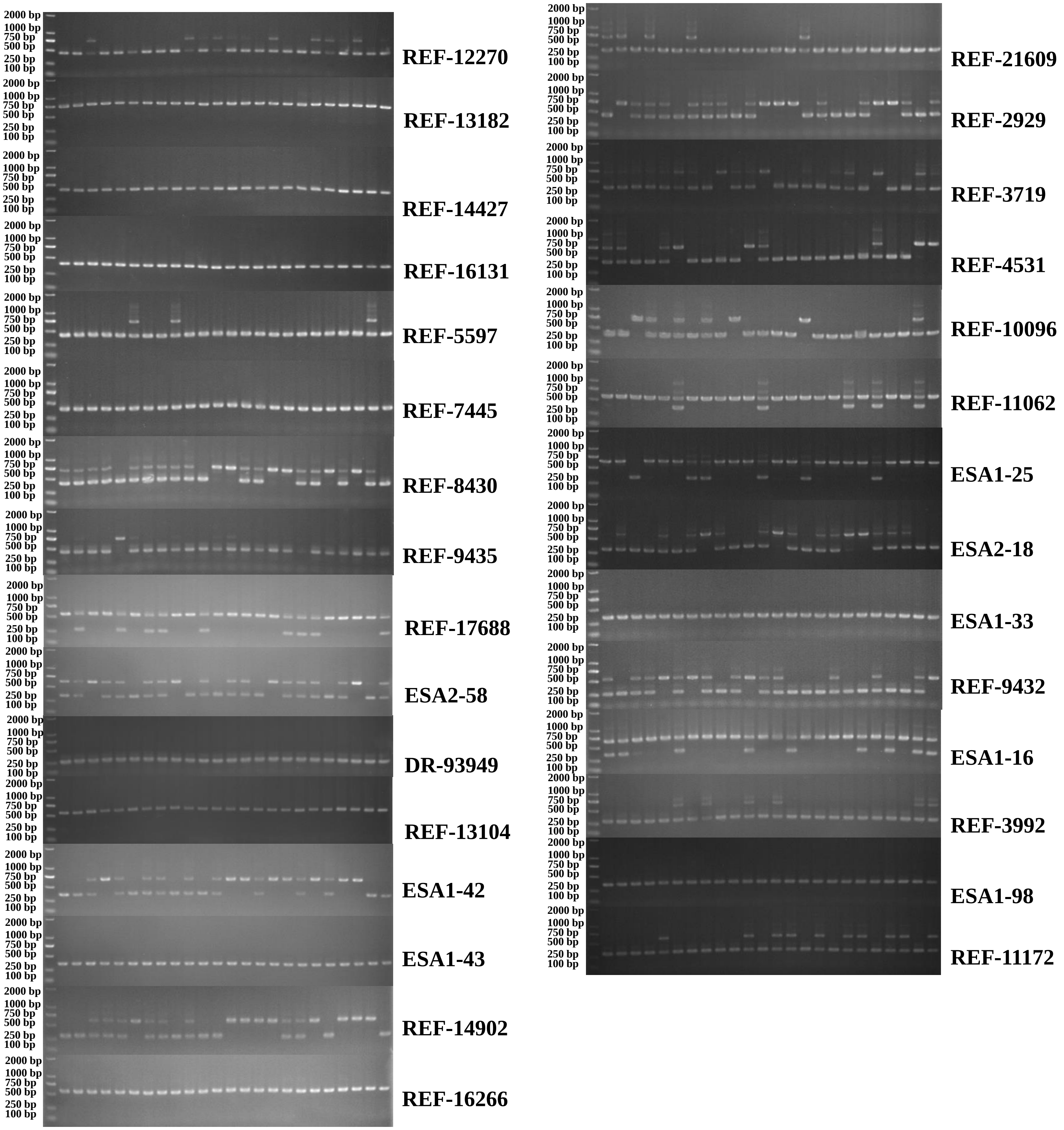

Wuzhishan pig

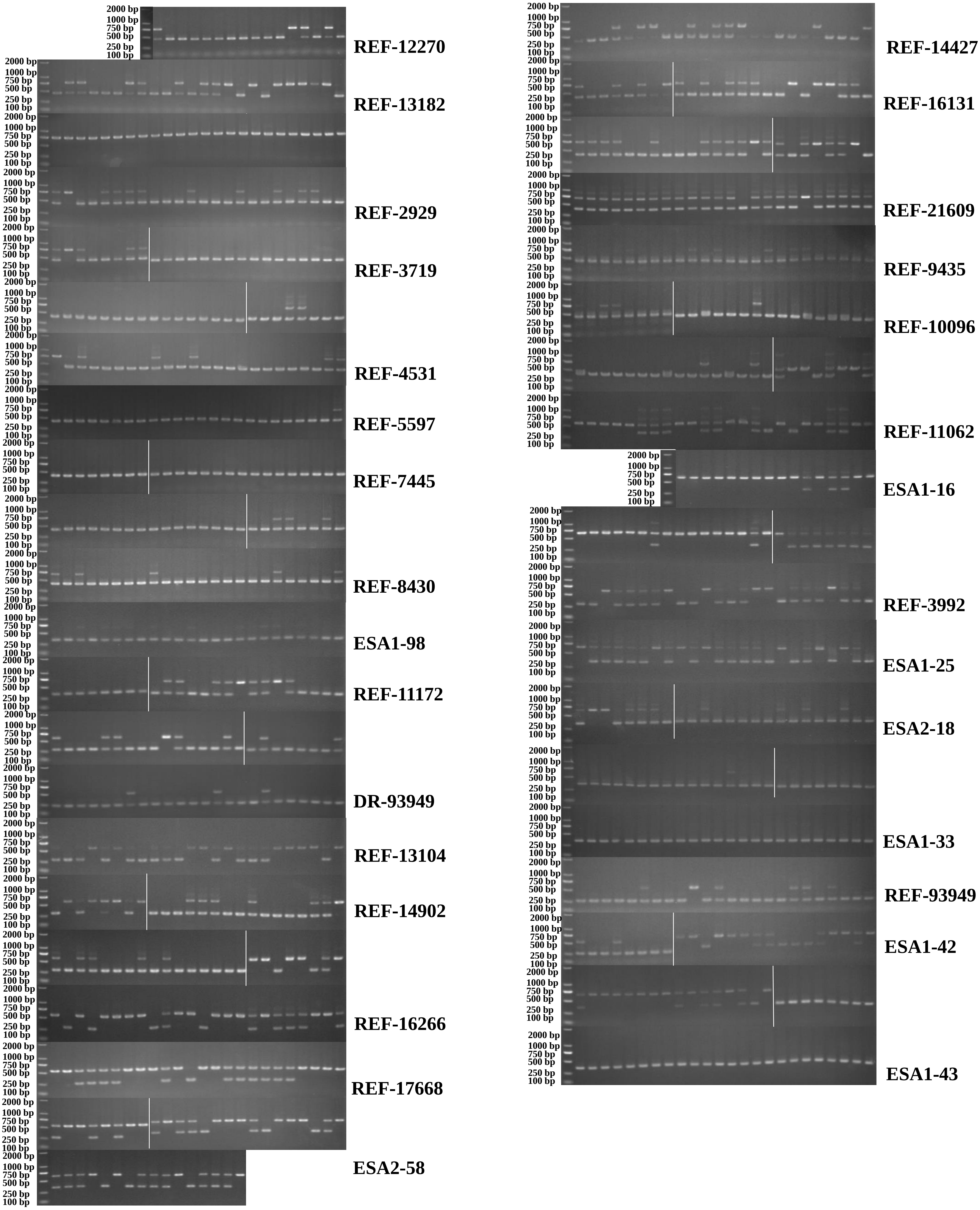

Sicilian black pig

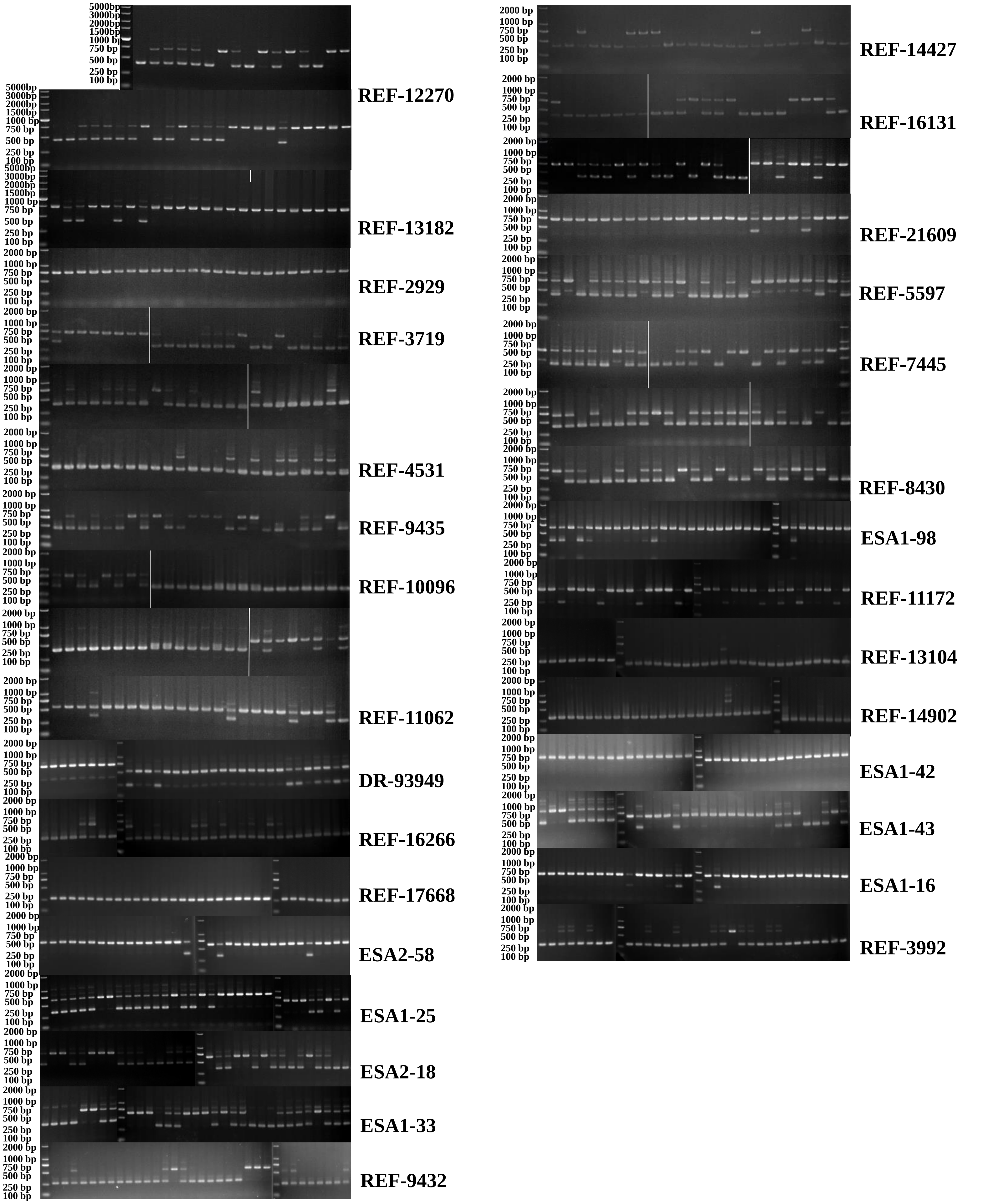

Landrace pig

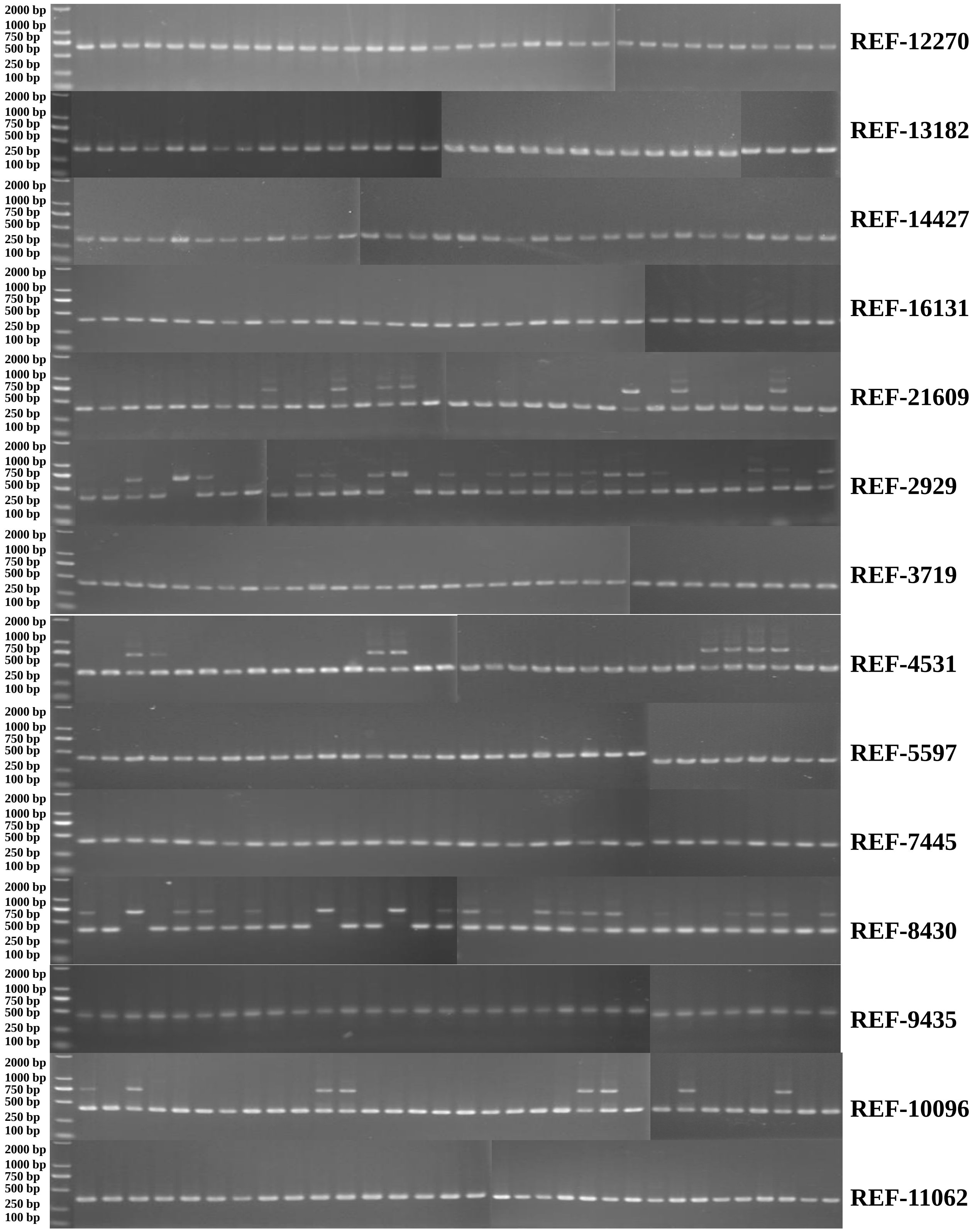

Landrace pig

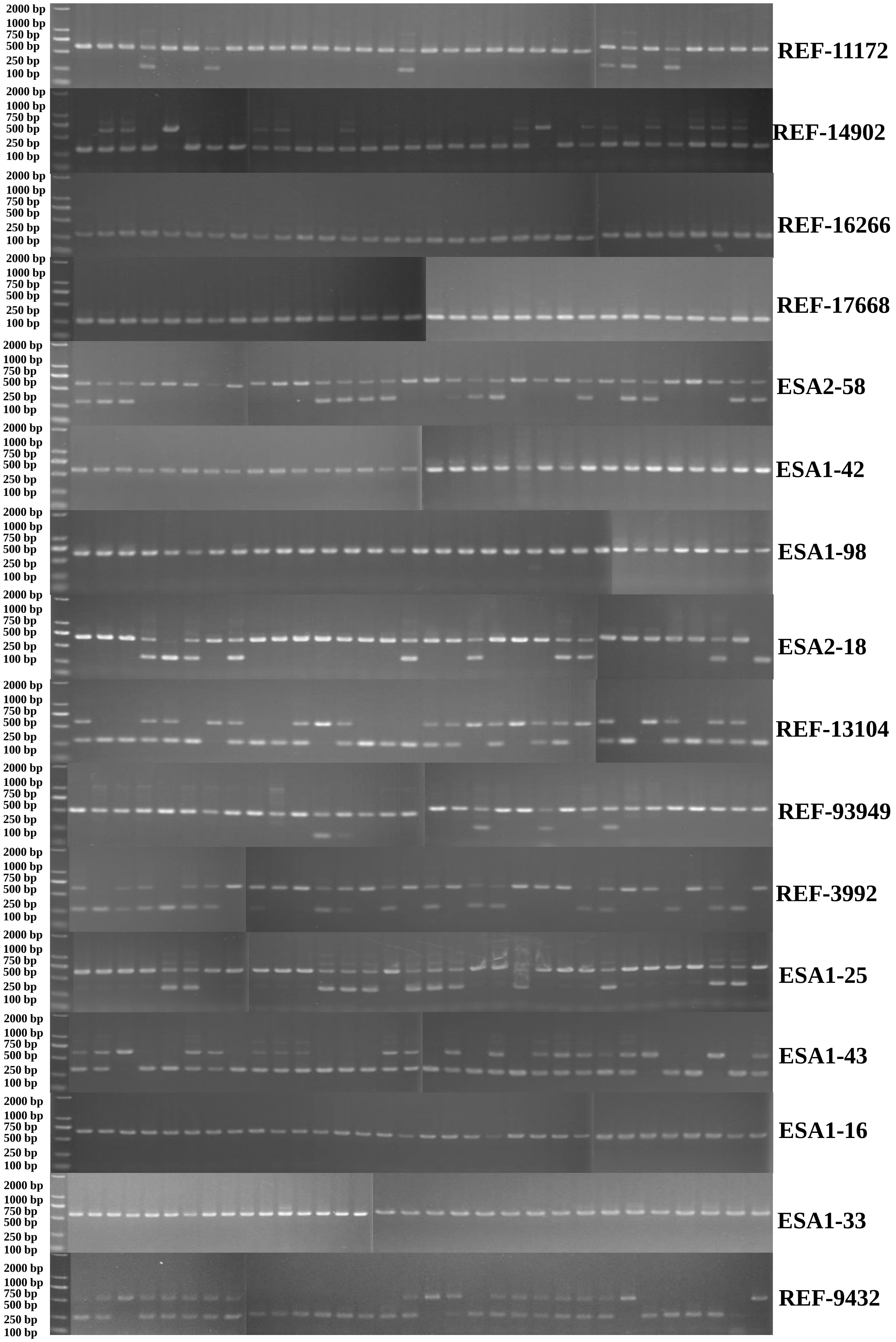

Supplement: Supplementary file 1 [file animals-11-01136-s001.zip › animals-1155464-revised- supplementary/Figure S1.pdf]
