# Supplementary material for: Genetic Diversity and Population Structures in Chinese Miniature Pigs Revealed by SINE Retrotransposon Insertion Polymorphisms, a New Type of Genetic Markers
Source: Animals (Basel). 2021 Apr 15;11(4):1136. doi: 10.3390/ani11041136 (PMC8071531; doi:10.3390/ani11041136)
Supplement: Supplementary file 1 [file animals-11-01136-s001.zip › animals-1155464-revised- supplementary/Table S1.docx]

Table S1 Primers of the 36 SINE RIP markers

| Marker number | Primer name | Primer sequence | Location | Tm/℃ | Predicted PCR product size (bp) |
| --- | --- | --- | --- | --- | --- |
| *1* | *REF-815* | 1. *TCAGCCTGTTTCTCTTGGTCA*   *R-TCTGGGTTTCCTCTGTGCAT* | [chr1:78702751-78703162](https://genome.ucsc.edu/cgi-bin/hgTracks?hgsid=1006608193_AkcsMR48aWBwkoy8ot8jSnTA30iA&db=susScr11&position=chr1:78702751-78703162&hgPcrResult=pack) | *58* | *412/696* |
| 2 | REF-12270 | F-CTTTTGTCCTTCACTGTTCATCA  R-TCTGCGTTGTGTCCACTCTA | [chr2:132104503-132104952](https://genome.ucsc.edu/cgi-bin/hgTracks?hgsid=1006608193_AkcsMR48aWBwkoy8ot8jSnTA30iA&db=susScr11&position=chr2:132104503-132104952&hgPcrResult=pack) | 58 | 450/713 |
| 3 | REF-13182 | F-GGAGGCAAAGGGAAAGAGTC  R-GCTCCCATTCCTGTTGTTGT | [chr3:74208760-74209181](https://genome.ucsc.edu/cgi-bin/hgTracks?hgsid=1006608193_AkcsMR48aWBwkoy8ot8jSnTA30iA&db=susScr11&position=chr3:74208760-74209181&hgPcrResult=pack) | 58 | 432/727 |
| 4 | REF-14427 | F-ACAACACAAGCCCCAAATGA  R-TGACTTTTCTGTGTTGGTCTTGT | [chr4:79318193-79318592](https://genome.ucsc.edu/cgi-bin/hgTracks?hgsid=1006608193_AkcsMR48aWBwkoy8ot8jSnTA30iA&db=susScr11&position=chr4:79318193-79318592&hgPcrResult=pack) | 58 | 400/650 |
| 5 | REF-16131 | F-TGCTTTTCTTCTGGGAGGTG  R-CAACGCTTGCCAGATTTTCT | [chr5:98261686-98262086](https://genome.ucsc.edu/cgi-bin/hgTracks?hgsid=1006608193_AkcsMR48aWBwkoy8ot8jSnTA30iA&db=susScr11&position=chr5:98261686-98262086&hgPcrResult=pack) | 58 | 432/732 |
| *6* | *REF-16684* | *F-CCCAAGTGGCTTATGTGTGG*  *R-TGCAGTACATCCTTGAGCCT* | [chr6:43833547-43833982](https://genome.ucsc.edu/cgi-bin/hgTracks?hgsid=1006608193_AkcsMR48aWBwkoy8ot8jSnTA30iA&db=susScr11&position=chr6:43833547-43833982&hgPcrResult=pack) | *58* | *432/722* |
| *7* | *REF-18327* | *F-CAGATGAGGTTGAGCTGTGC*  *R-GTTTGTCCCTCTCTCCCACT* | [chr7:59713663-59715467](https://genome.ucsc.edu/cgi-bin/hgTracks?hgsid=1006608193_AkcsMR48aWBwkoy8ot8jSnTA30iA&db=susScr11&position=chr7:59713663-59715467&hgPcrResult=pack) | *58* | *411/617* |
| *8* | *REF-19717* | *F-ACACAGGTTTGAGAGCAGAC*  *R-GTTGACCTTCTGCCTCATGG* | [chr8:85085731-85086164](https://genome.ucsc.edu/cgi-bin/hgTracks?hgsid=1006608193_AkcsMR48aWBwkoy8ot8jSnTA30iA&db=susScr11&position=chr8:85085731-85086164&hgPcrResult=pack) | *58* | *434/706* |
| 9 | REF-21609 | F-GAATGCCACTTTCCCCACAA  R-TCAGTGAGTAGGTGGCAGAG | [chr9:122993311-122993721](https://genome.ucsc.edu/cgi-bin/hgTracks?hgsid=1006608193_AkcsMR48aWBwkoy8ot8jSnTA30iA&db=susScr11&position=chr9:122993311-122993721&hgPcrResult=pack) | 58 | 411/693 |
| 10 | REF-2929 | F-CCAACTGCATGCTCTTCCAG  R-TCCTGATTATCTTGGAAATGGCT | [chr10:59802500-59802944](https://genome.ucsc.edu/cgi-bin/hgTracks?hgsid=1006608193_AkcsMR48aWBwkoy8ot8jSnTA30iA&db=susScr11&position=chr10:59802500-59802944&hgPcrResult=pack) | 58 | 445/739 |
| 11 | REF-3719 | F-TTCTCTTCCCTTCCTGACCG  R-CATGTTGGTACCCCTCCCAT | [chr11:60869832-60870233](https://genome.ucsc.edu/cgi-bin/hgTracks?hgsid=1006608193_AkcsMR48aWBwkoy8ot8jSnTA30iA&db=susScr11&position=chr11:60869832-60870233&hgPcrResult=pack) | 58 | 402/711 |
| 12 | REF-4531 | F-AGCTTTCCCTCTTTCTCCGT  R-CCGTCCATATTGCCAAGTCG | [chr12:47951148-47951587](https://genome.ucsc.edu/cgi-bin/hgTracks?hgsid=1006608193_AkcsMR48aWBwkoy8ot8jSnTA30iA&db=susScr11&position=chr12:47951148-47951587&hgPcrResult=pack) | 58 | 440/737 |
| 13 | REF-5597 | F-AACCAAAGCAGTGTTCAGGG  R-AGGGTTTGGGATGATGATGGT | [chr13:106226070-106226486](https://genome.ucsc.edu/cgi-bin/hgTracks?hgsid=1006608193_AkcsMR48aWBwkoy8ot8jSnTA30iA&db=susScr11&position=chr13:106226070-106226486&hgPcrResult=pack) | 58 | 417/710 |
| 14 | REF-7445 | F-TCTAGCTTTTCTTACCATTGGCT  R-ATCCAGTGGCTAGGCTAGAC | [chr14:125956551-125956960](https://genome.ucsc.edu/cgi-bin/hgTracks?hgsid=1006608193_AkcsMR48aWBwkoy8ot8jSnTA30iA&db=susScr11&position=chr14:125956551-125956960&hgPcrResult=pack) | 58 | 410/673 |
| 15 | REF-8430 | F-ACCACACAAGGCACATTTTG  R-GGCCTCTTTATTCACCCTCCT | [chr15:97783397-97783813](https://genome.ucsc.edu/cgi-bin/hgTracks?hgsid=1006608193_AkcsMR48aWBwkoy8ot8jSnTA30iA&db=susScr11&position=chr15:97783397-97783813&hgPcrResult=pack) | 58 | 417/631 |
| 16 | REF-9435 | F-GCCTGTGTCAGTACTTCATTCA  R-TGGGGTTAACAGATACACACTAC | [chr16:55631429-55631871](https://genome.ucsc.edu/cgi-bin/hgTracks?hgsid=1006608193_AkcsMR48aWBwkoy8ot8jSnTA30iA&db=susScr11&position=chr16:55631429-55631871&hgPcrResult=pack) | 58 | 443/669 |
| 17 | REF-10096 | F-CTCGCCCCTTACTTCAGACA  R-AACCATCACCACTGAACCCC | [chr17:30484896-30485305](https://genome.ucsc.edu/cgi-bin/hgTracks?hgsid=1006608193_AkcsMR48aWBwkoy8ot8jSnTA30iA&db=susScr11&position=chr17:30484896-30485305&hgPcrResult=pack) | 58 | 410/672 |
| 18 | REF-11062 | F-AGTCTCCCACTCACATTGCC  R-CCTCTGAGCTGCTCTTCCTT | [chr18:50578007-50578406](https://genome.ucsc.edu/cgi-bin/hgTracks?hgsid=1006608193_AkcsMR48aWBwkoy8ot8jSnTA30iA&db=susScr11&position=chr18:50578007-50578406&hgPcrResult=pack) | 58 | 400/690 |
| 19 | ESA1-98 | F-GAGCATTGCACCTGCACTTA  R-CACTTCTTGCAAAACACTGTAGG | chr1:119154448-119155108 | 58 | 404/661 |
| 20 | REF-11172 | F-AGCTGAACTGGGTGTTTTTG  R-CACATCTGGTCCAGGATAGAC | chr2:8443537-8443836 | 58 | 300/584 |
| 21 | REF-13104 | F-CAGAAACTTGTGCCTCCAAT  R-TGGACTAGCAAGAATCGTCA | [chr3:67608258-67608555](https://genome.ucsc.edu/cgi-bin/hgTracks?hgsid=1006608193_AkcsMR48aWBwkoy8ot8jSnTA30iA&db=susScr11&position=chr3:67608258-67608555&hgPcrResult=pack) | 58 | 298/570 |
| 22 | REF-14902 | F-GCCAATCTGAAAAGGCTACA  R-TACATTTGTTGCACCGACAC | chr4:122415857-122416162 | 58 | 306/593 |
| *23* | *DR-68328* | *F-TGCGCATGGAATATCAATTA*  *R-TTTGGTGTTCATTGGTGATG* | *chr5: 63213287-63213842* | *58* | *258/556* |
| 24 | REF-16266 | F-TCCGCCCATCTGAATAATAA  R-GCATTACCTTTGGTGAGAGG | chr6:9549227-9549524 | 58 | 298/555 |
| 25 | REF-17668 | F-CCTTTGCCATTTCTCCAGTA  R-AGTTACAGACCCGGCTTACC | chr7:1569064-1569342 | 58 | 279/561 |
| 26 | ESA2-58 | F-GGAGCCTTTCTGTGTTTCCA  R-GAGCAAGAAGAGGGGAGGTAA | chr8:28073574-28074197 | 58 | 361/624 |
| 27 | DR-93949 | F-GAAGAATGAGAGCACCTCCA  R-AAAATGGCCTTTGTCCTCTT | chr9:122606755-122607292 | 58 | 276/538 |
| *28* | *DR-13618* | *CTGTTCAGGGCTCAAAAGAA*  *ACGGGGAAATTAACCAAAAA* | *chr10: 68538669-68539215* | *58* | *282/547* |
| 29 | ESA1-16 | F-GTCCCCAAAACAGTGTGGAG  R-ACTCGGGGAACTTCCTTCAT | chr11:68768136-68768788 | 58 | 395/653 |
| 30 | REF-3992 | F-ATGTACCCTCCCCTCAAATC  R-TACCCCAAACACCAAAACAT | chr12:3226907-3227211 | 58 | 305/603 |
| 31 | ESA1-25 | F-GGTGCCCTACAATGCCTCTA  R-CCCTCTGTGACCTTGGAAAA | chr13:45743481-45744130 | 58 | 393/650 |
| 32 | ESA2-18 | F-AGTCCTGTGCTGGGAAGTTG  R-TTCCACTTTGCAGCATTTTG | chr14:107388631-107389282 | 58 | 385/652 |
| 33 | ESA1-33 | F-TCTGATAGGCGCTGGATCTT  R-GGCCCTGGAGCTTCTACAT | chr15:104894531-104895172 | 58 | 385/642 |
| 34 | REF-9432 | F-TGGCTGTAGGATTGTGACTG  R-TTGGTGAAAATCCAAAACCT | chr16:55338119-55338391 | 58 | 273/495 |
| 35 | ESA1-42 | F-TTGTCCAAGTGGTTTTGTGG  R-TGCCAAGTTCATCTTCAGGA | chr17:53364230-53364880 | 58 | 394/651 |
| 36 | ESA1-43 | F-CAATGCAATGCTGTGGAATC  R-ATAGACAAGGGAGGGGAGGA | chr18:37184460-37185105 | 58 | 392/646 |

Note: The marker in italics is that the amplification result is not easy to determine the genotype or there is no polymorphism in all miniature pig populations and were not be used in further detection.
