# Supplementary material for: Genetic Diversity and Population Structures in Chinese Miniature Pigs Revealed by SINE Retrotransposon Insertion Polymorphisms, a New Type of Genetic Markers
Source: Animals (Basel). 2021 Apr 15;11(4):1136. doi: 10.3390/ani11041136 (PMC8071531; doi:10.3390/ani11041136)
Supplement: Supplementary file 1 [file animals-11-01136-s001.zip › animals-1155464-revised- supplementary/Tbale S3.docx]

Table S3 Comparison of population genetic parameter results based on RIPs, microsatellites and SNPs.

| Genetic parameter | Bama pigs | | |  | Tibetan pigs | | |  | Wuzhishan pigs | | |
| --- | --- | --- | --- | --- | --- | --- | --- | --- | --- | --- | --- |
| Marker type | SINE RIP | SNP | MICRO |  | SINE RIP | SNP | MICRO |  | SINE RIP | SNP | MICRO |
| PIC | 0.1777 | - | 0.5469[55] |  | 0.1885 | - | 0.7663[55]/0.696[58] |  | 0.2238 | - | 0.7069[56]/0.84[57]/0.653[58] |
| Ho | 0.2097 | 0.21[59] | - |  | 0.2431 | 0.24[59] | 0.515[58] |  | 0.2698 | 0.25[59] | 0.498[58] |
| He | 0.2252 | - | 0.5428[55] |  | 0.2378 | - | 0.746[58] |  | 0.2814 | - | 0.5478[56]/0.707[58] |

Note: PIC: polymorphic information content, Ho: observed heterozygosity, He: expected heterozygosity. MICRO: microsatellite.
